# Expression of LGR-5, MSI-1 and DCAMKL-1, putative stem cell markers, in the early phases of 1,2-dimethylhydrazine-induced rat colon carcinogenesis: correlation with nuclear β-catenin

**DOI:** 10.1186/1471-2407-13-48

**Published:** 2013-02-01

**Authors:** Angelo Pietro Femia, Piero Dolara, Maddalena Salvadori, Giovanna Caderni

**Affiliations:** 1 Department of Pharmacology, University of Florence, 6 Viale Pieraccini, 50139, Florence, Italy

**Keywords:** Stem cells, Colon carcinogenesis, Precancerous lesions

## Abstract

**Background:**

Colon cancer stem cells may drive carcinogenesis and account for chemotherapeutic failure. Although many markers for these cells have been proposed, there is no complete agreement regarding them, nor has their presence in the early phases of carcinogenesis been characterized in depth.

**Methods:**

The expression of the putative markers LGR-5 (leucine-rich-repeat-containing G-protein-coupled receptor 5), MSI-1 (Musashi-1) and DCAMKL-1 (doublecortin and calcium/calmodulin-dependent protein kinase-like-1) was studied in normal colon mucosa (NM), in the precancerous lesions Mucin Depleted Foci (MDF) and in macroscopic tumours (adenomas) of 1,2-dimethylhydrazine-treated rats. Co-localization between these markers and nuclear β-catenin (NBC), an attributed feature of cancer stem cells, was also determined. Moreover, since PGE_2_ could increase NBC, we tested whether short-term treatment with celecoxib, a COX-2 inhibitor (2 weeks, 250 ppm in the diet) could reduce the expression of these markers.

**Results:**

LGR-5 expression in NM was low (Labelling Index (LI): 0.22±0.03 (means±SE)) with positive cells located mainly at the base of the crypts. Compared to NM, LGR-5 was overexpressed in MDF and tumours (LI: 4.7±2.0 and 2.9±1.0 in MDF and tumours, respectively, P<0.01 compared to NM). DCAMKL-1 positive cells, distributed along the length of normal crypts, were reduced in MDF and tumours. Nuclear expression of MSI-1, located mainly at the base of normal crypts, was not observed in MDF or tumours. In both MDF and tumours, few cells co-expressed LGR-5 and NBC (LI: 1.0±0.3 and 0.4±0.2 in MDF and tumours, respectively). Notwithstanding the lower expression of DCAMKL-1 in tumours, the percentage of cells co-expressing DCAMKL-1 and NBC was higher than in NM (LI: 0.5±0.1 and 0.04±0.02 in tumours and NM, respectively). MSI-1 and NBC co-localization was not observed. Celecoxib did not reduce cells co-expressing LGR-5 and NBC.

**Conclusions:**

Based on its prevalent localization at the base of normal crypts, as expected for stem cells, and on the overexpression in precancerous lesions and tumours, we support LGR-5, but not MSI-1 or DCAMKL-1, as putative neoplastic stem cell marker. In both MDF and tumours, we identified LGR-5-positive cells co-expressing NBC which could be a subpopulation with the highest stem cell features.

## Background

Cancer stem cells are increasingly considered as those cells responsible for colon carcinogenesis as well as the reason for the failure of chemotherapy [1-4]. Therefore, an understanding of their biology and their involvement in the various phases of carcinogenesis is critical to planning both new chemopreventive and anti-tumour strategies. While many studies have focused on the identification of cancer stem cells in advanced tumours and on their resistance to cytotoxic drugs, their involvement in the very early phases of carcinogenesis has been less studied.

Colon carcinogenesis induced in the rat by azoxymethane or 1,2-dimethylhydrazine (DMH), is a model mimicking the sequence of the histopathological and molecular alterations observed in human pathology [5,6]. In this experimental setting, the study of precancerous lesions such as Mucin Depleted Foci (MDF) can allow the identification of the earliest alterations preceding colon tumour development [6]. We have previously shown that MDF, like tumours, harbour mutations in the *Apc* and *Ctnnb1* genes, members of the Wnt pathway and show activation of this signaling [7,8], with, at least some cells, expressing β-catenin in the nucleus [7]. Accordingly, Wnt activation can be demonstrated as intracellular, notably nuclear, accumulation of β-catenin which in the nucleus can activate gene transcription [9]. Interestingly, it has been reported that although *APC* or β-catenin mutations can be present in all the cells of a tumour, not all these cells display nuclear β-catenin [10,11]. One possible explanation for this “paradox” [12], is that other factors could contribute to β-catenin nuclear translocation. It has been suggested that local mediators such as COX-derived PGE_2_[13] or HGF (hepatocyte growth factor) present within the microenvironment could activate the Wnt pathway [11]. Moreover, experimental studies have reported that cancer cells with stemness ability are those showing high activity of the Wnt pathway (i.e. nuclear expression of β-catenin) [11], thus suggesting that the expression of this marker can aid in the identification of stem cells.

In recent years, many specific epitopes have been suggested as markers of stem cells and, probably, cancer stem cells, such as CD44, Musashi-1 (MSI-1), CD133, CD166, DCAMKL-1 (doublecortin and calcium/calmodulin-dependent protein kinase-like-1), ALDH-1 (aldehyde dehydrogenase 1), LRIG (leucine-rich repeats and immunoglobulin-like domains-1) and LGR-5 (leucine-rich-repeat-containing G-protein-coupled receptor 5) [14-21]. If these markers identify the same population of cells (i.e. the true stem cells), one would expect an almost complete overlapping of them. On the contrary, some of these markers distinguish different populations of cells [22] suggesting the existence of different stem cell populations (quiescent or active), or, perhaps, the need of more specific markers.

We reported recently that colon carcinomas from DMH-induced rats which show constitutive activation of the Wnt pathway, also overexpress the gene for *LGR-5*[23]*,* putative stem cell marker in the intestine of mice and humans and target gene of Wnt. On the contrary, genes of other proposed stem cell markers, such as *Dcamkl-1*, *Msi-1* and *Prom-1* (CD133), were down-regulated in DMH-tumours [23]. The expression of these markers at the protein level as well as the identification and localization of the cells expressing them in the early phases of carcinogenesis has not been yet studied.

Based on these premises, in order to characterize the expression of putative stem markers during the early phases of carcinogenesis, we studied the expression of LGR-5, MSI-1, DCAMKL-1, CD133 and ALDH1-A1 in both MDF and tumours by immunohistochemistry. Since the combination of two markers could improve the identification of putative neoplastic stem cells , we also studied the co-localization of some of the above markers with nuclear β-catenin.

It has been suggested that PGE_2_ could enhance Wnt activity, i.e. favor the translocation of β-catenin in the nucleus [13] conferring stemness features [24], so we also tested whether a short-term treatment with celecoxib, a selective COX-2 inhibitor, could reduce cells expressing these putative stem cell markers in tumours from DMH-induced rats.

## Methods

### Animals and induction of carcinogenesis

Male F344 rats, 4–5 weeks old, were obtained from Nossan (Milan, Italy) and were housed according to the European Union Regulations on the Care and Use of Laboratory Animals [25]; approval of the protocol was received by the Italian Ministry of Health (ID approval 141/2008-B). Rats were fed throughout the study with a High Fat (HF) diet based on the AIN-76 diet as reported [26]. At 6–7 weeks of age rats were treated twice, one week apart, with subcutaneous injections of DMH (150 mg/kg × 2 times) to induce colon carcinogenesis [26]. A group of animals (n=4) was sacrificed after 15 weeks from DMH to harvest the pre-neoplastic lesions MDF as described [8]. Another group of animals (n=9) continued the same dietary regimen until 24 weeks after DMH administration, a time at which macroscopic tumours (adenomas) were already developed [7,8]. At this time point, animals were divided into two experimental groups as follows: 1) rats (n=4) continued on the same experimental diet (controls); 2) rats (n=5) were administered a diet supplemented with celecoxib (Celebrex, Pfizer) at 250 ppm. All animals were sacrificed with CO_2_ asphyxiation 26 weeks after DMH (total treatment period with celecoxib: 2 weeks).

### Collection of the samples

After animals were sacrificed, the colon was removed, washed with saline, longitudinally opened, pinned on a polystyrene board and fixed in buffered formalin at room temperature for at least 3 h. Colons from the first group of animals (sacrificed 15 weeks after DMH) were stained for 10 min in 1% Alcian blue (AB) in 3% acetic acid followed by 15 sec of a 0.5% water solution of neutral red (NR) [8], to identify MDF under the microscope. MDF were marked laterally with permanent ink, dissected together with a portion of apparently normal mucosa and embedded in paraffin, to be processed for immunohistochemistry as described below. At sacrifice, colons from the second group of animals (sacrificed after 26 weeks after DMH) were examined for the presence of macroscopic tumours and number and size (measured by a calliper) were registered. Colons were then fixed in buffered formalin for at least 3 hours and tumours were excised along with a fragment of adjacent normal mucosa and paraffin-embedded for subsequent immunohistochemistry analysis. Longitudinal sections (4 μm thick) containing the lesion (MDF or tumour) together with the surrounding normal mucosa were mounted on electrostatic-treated slides (Superfrost® Plus, Medite, Italy) to be processed for immunohistochemistry as described below. Given the relative small number of slides that can be obtained from a rat lesion, it was not always possible to test each sample (MDF or tumour) with all the different antibodies and therefore the number of samples for each marker is not always the same.

### Bright-field immunohistochemistry

The sections were processed as described [27] using the following primary antibodies: LGR-5/GPR49 (rabbit, monoclonal, Abcam, ab75850); MSI-1 (rabbit, monoclonal, Abcam, ab52865); DCAMKL-1 (rabbit, polyclonal, Abcam, ab31704), CD133 (rabbit, polyclonal, Abnova, PAB12663); ALDH1-A1 (rabbit, polyclonal, Abcam, ab23375); β-catenin (mouse, monoclonal, BD Transduction Laboratories, catalogue number 610154). Antibodies were diluted in PBS containing 1% bovine serum albumin (BSA) and incubated as follows: LGR-5/GPR49 1:250, 1h at room temperature (RT); MSI-1 1:75, 2h at RT; DCAMKL-1 1: 200, 1 h at RT; β-catenin 1:1000, over night at 4°C. The slides were then rinsed twice in PBS, covered with Biotinylated Goat Anti-Polyvalent as the secondary antibody (LAB Vision Corporation, CA, USA). Immunohistochemical staining was performed using the streptavidin-biotin immunoenzymatic antigen detection system (Ultravision Large Volume Detection System Anti-Polivalent, HRP (LAB Vision Corporation, CA, USA) followed by reaction with 3,3’-diaminobenzidine (Liquid DAB Substrate Pack, concentrated; Biogenex, CA, USA). The slides were weakly counterstained with Harris’ hematoxylin and observed under a microscope to evaluate the labelling with each antibody identified by brown staining. Negative controls in which the primary antibody was omitted were performed in each experiment. Reactivity evaluation in the normal mucosa was carried out in crypts separated from the lesion by an area occupied by at least three crypts. The same evaluation was carried out for the MDF or tumour present in the same slide.

Labelling Index (LI) was quantified by determining the number of labelled cells/total number of cells counted × 100. Evaluation was performed at 400 × magnification. The distribution of the labelled cells along the crypts of the normal mucosa, was evaluated by recording the number of labelled cells in the three compartments of the crypt visible in longitudinally sectioned crypts, ideally divided into three equal parts: lower, mid and upper.

### Fluorescence immunohistochemistry

Paraffin-embedded sections from MDF or tumours together with the surrounding normal mucosa were used for co-localization of LGR-5, MSI-1 or DCAMKL-1 with nuclear β-catenin by immunofluorescence. Sections were dewaxed with xylene, hydrated through gradations of ethanol reaching distilled water. Sections were then immersed in citric acid buffer (pH 6.0) and microwaved for 3 cycles (5 min each). The slides were then allowed to cool for 30 min while immersed in citrate buffer, washed three times with PBS containing 0.05% Tween 20. Sections were then incubated for 1 h with blocking solution containing 5 mg/ml BSA and 5% Normal Goat Serum in PBS, pH 7.4 and then incubated with one of the primary antibodies diluted in blocking solution according to their incubation time as described above. Sections were then incubated for 2 h in the dark with the fluorescent secondary antibody (Alexa Fluor® 488 Donkey anti-rabbit IgG, Molecular Probes, Life Technologies) diluted 1:400 in blocking solution. Thereafter, sections were incubated with the primary antibody against β-catenin (1:1000 in PBS, over night at 4°C) and then incubated for 2 h in the dark with the fluorescent secondary antibody (Alexa Fluor® 594 Rabbit anti-mouse IgG, Molecular Probes, Life Technologies) diluted 1:400 in blocking solution. Sections were finally counterstained with 4’,6-diamidino-2-phenylindole (DAPI, 1μg/ml) (Vector Laboratories). The analysis of negative controls (omission of primary antibody) were simultaneously performed to exclude the presence of non-specific immunofluorescence staining. An Olympus BX40 microscope coupled to analySIS^B Imaging Software (Olympus) was used to observe and acquire images from the examined specimens.

### Quantification of the co-localization of LGR-5, MSI-1 or DCAMKL-1 with nuclear β-catenin by immunofluorescence technique

For each sample (MDF or tumours together with the surrounding normal mucosa), random fields were analyzed and images acquired at 400x magnification. Pictures were then displayed on a computer monitor and identification of cells co-expressing nuclear β-catenin and LGR-5 (or MSI-1 and DCAMKL-1) (co-localizing cells) was performed by a complete scanning of the image. The total number of epithelial cells was also registered for each image. The percentage of co-localizing cells, expressed as Labelling Index (LI), was calculated as the ratio between co-localizing cells/ total cells in the field ×100.

### Statistical analysis

Differences between the labelling index (LI) of the lesions (MDF or tumours) and that of the surrounding normal mucosa (NM) present in the same section and harvested from the same animal were analysed using paired t-test; on the contrary, differences between the lesions (MDF and/or tumours) harvested from different animals were analysed with unpaired t-test. In both cases t-tests were two sided. The differences between the distribution of labeled cells along the crypt for the different markers were evaluated with chi square analysis. Differences were considered significant when P was < 0.05.

## Results

### Expression of LGR-5, MSI-1, DCAMKL-1, CD133 and ALDH1-A1 in normal mucosa, MDF and tumours (bright field immunohistochemistry)

**Figure 1:**
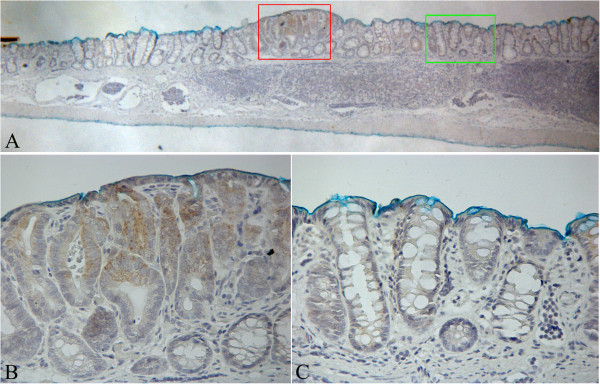
**Histological section of a Mucin Depleted Focus (MDF) processed by immunohistochemistry with the LGR-5 antibody.** Panel **A**: Low power magnification of a MDF (red inset) with its surrounding normal mucosa (original magnification 40x). Panel **B**: higher magnification of the MDF (red inset in panel A) in which an over-expression of LGR-5 is observed. Panel **C**: higher magnification of the normal mucosa (green inset of panel A). Original magnification 400x.

**Figure 2:**
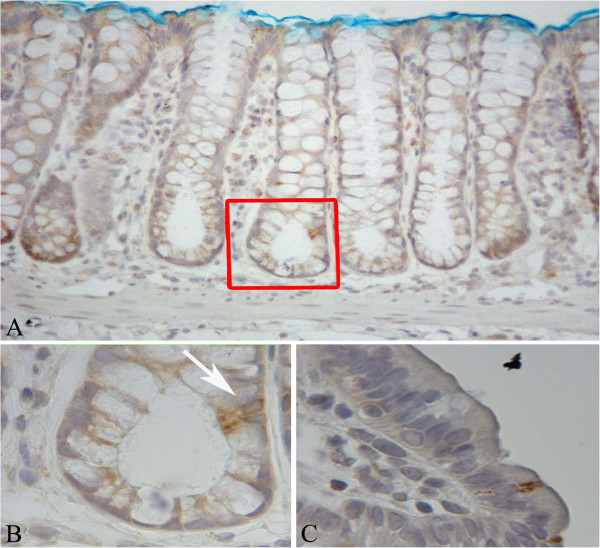
**Expression of LGR-5 in the normal colonic mucosa.** Panel **A**: longitudinally cut crypts in which positive cells in the lower compartment are visible (within the red inset); original magnification 200x. Panel **B**: magnification of the red inset in panel A; original magnification 1000x. Panel **C**: single stained cell in the luminal compartment of the crypt; original magnification 400x.

**Figure 3:**
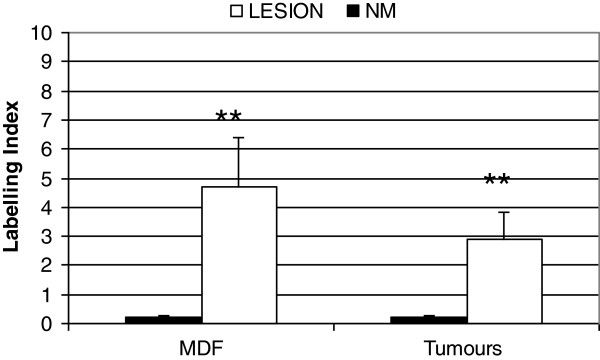
**LGR-5 expression (as labelling index (LI)) in MDF and tumours (white bars) and in their respective surrounding mucosa (NM: black bars).** Values are means + SE (n=13 and 15 in MDF and tumours, respectively). **: P value <0.01 when compared with the corresponding normal mucosa.

**Figure 4:**
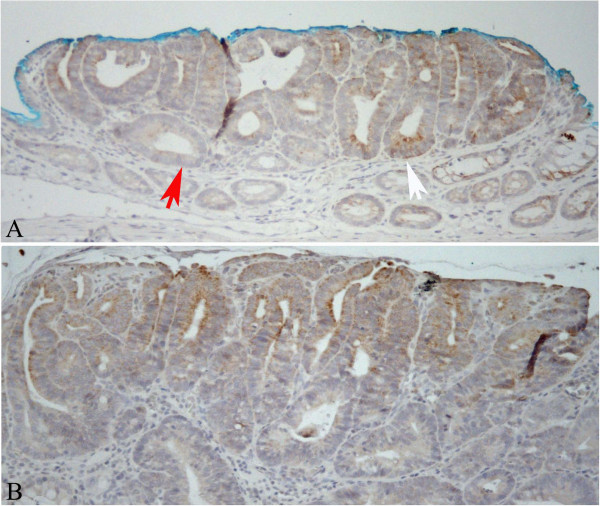
**Expression of LGR-5 in two different tumours.** Panel **A**: heterogeneous expression of LGR-5 inside a tumour in which positive (white arrow) and negative (red arrow) areas are present. Panel **B**: LGR-5 is expressed toward the luminal part. Original magnification 400x.

Analysis of LGR-5 expression was carried out in 13 MDF and 15 tumours as well in the adjacent normal mucosa (NM). On the whole, LGR-5 expression was absent in most of the colon crypts in the normal mucosa (Figure 1, panels A and C). However, positive cells were also observed, located mostly in the lower third of the crypts (Figure 2, panels A and B), and, more rarely, in the luminal compartment (Figure 2, panel C). Some crypts of the normal mucosa showed a diffuse staining at the lower and middle compartments of the crypt (data not shown). The labeling index in the normal mucosa was 0.22±0.03 (means±SE; n=28) (Figure 3). The distribution of labelled cells along the crypt showed that most of them were located in the lower compartment (69% of the total labelled cells), while only 18% were in the middle region and 13% of the total labelled cells were located in the upper compartment. The labeling index in the MDF (n=13) and in tumours (n=15) showed (Figure 3) that the expression of LGR-5 was significantly increased (P<0.01) in both lesions compared with the surrounding normal mucosa (Figures 1, 4). The difference between the labeling index in MDF and tumours was not statistically significant (P=0.38). Staining in both MDF and tumours was heterogeneous, with positive areas and moderate or negative areas in the same lesion (Figure 4, panel A); in some cases, the staining was more evident in the upper compartment of the precancerous lesion or tumour (Figure 4, panel B).

**Figure 5:**
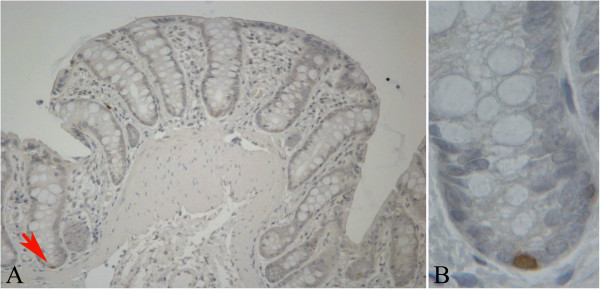
**Expression of MSI-1 in histological sections of normal colonic mucosa processed by immunohistochemistry.** Panel **A**: single nuclear stained cell at the base of one crypt (red arrow). Panel **B**: magnification of the area containing the stained cell in panel A. Original magnification 400x.

**Figure 6:**
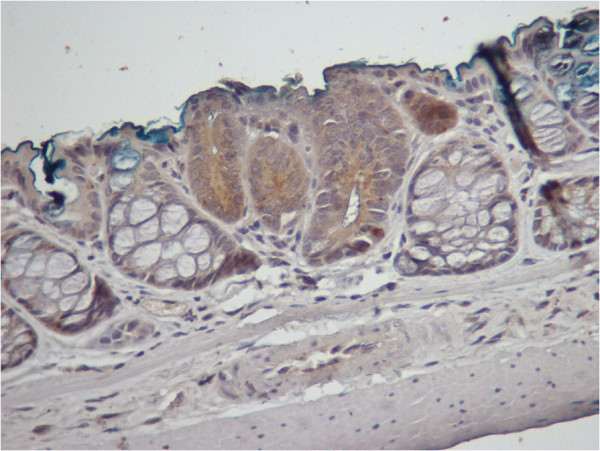
**Expression of MSI-1 in a histological section of a MDF processed by immunohistochemistry.** The lesion shows a diffuse cytoplasmic staining, not present in the normal mucosa. Original magnification 400x.

Expression of MSI-1 was determined in 7 MDF and 6 tumours and their surrounding normal mucosa. In the normal mucosa, we observed intensely stained cells (in the nucleus or in both nucleus and cytoplasm) (Figure 5). The labeling index was 0.17±0.03 (mean±SE, n=13; total cells counted 3782±462; mean±SE). The distribution of labelled cells along the crypt was similar to that observed for LGR-5 showing that most of them were located in the lower compartment (67% of the total labelled cells), while only 24% were in the middle and 9% in the upper compartment. Intensely stained cells were not present in either MDF or tumours. However, in 2 out of 7 MDF, we observed a diffuse cytoplasmic staining not present in the surrounding normal mucosa (Figure 6). A similar, although weaker, cytoplasmic staining was also observed in 2 out of 6 tumours analyzed (data not shown). The remaining MDF and tumour samples were completely negative.

**Figure 7:**
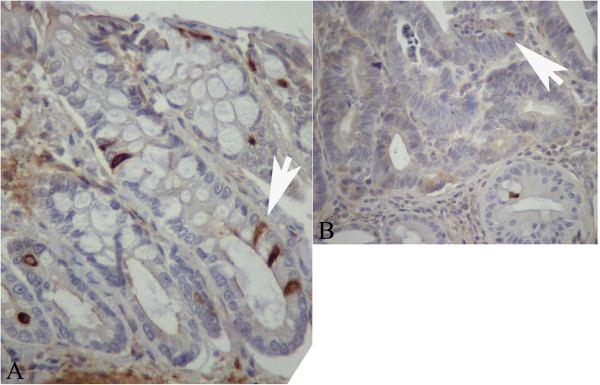
**Expression of DCAMKL-1 in histological sections of a normal colonic mucosa and a tumour.** Panel **A**: Normal mucosa; stained cells are distributed along the crypt. Panel **B**: Tumour. Examples of stained cells are indicated by white arrows. Original magnification 400x.

**Figure 8:**
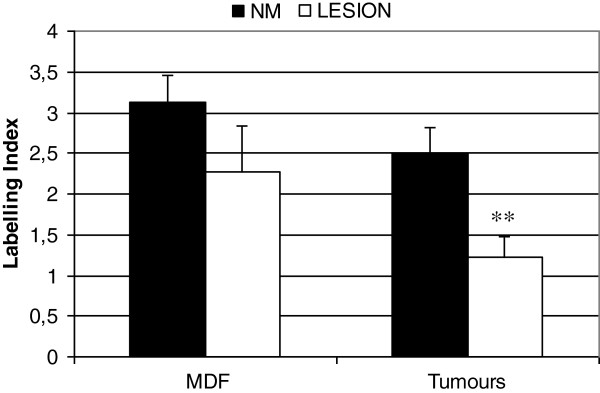
**DCAMKL-1 expression (as labelling index (LI)) in the preneoplastic lesions MDF, tumours (white bars) and in their respective surrounding mucosa (NM: black bars).** Values are means + SE (n=14 and 6 in MDF and tumours, respectively). **: P value <0.01 when compared with the corresponding normal mucosa.

DCAMKL-1 expression was evaluated in 14 MDF, 6 tumours and their surrounding normal mucosa. In normal crypts, positive cells were distributed along the entire length of the crypt, with only a slight prevalence in the lower part (Figure 7A). There were 47%, 27% and 26% of labeled cells in the lower, mid and upper compartments, respectively, a distribution different from that observed for LGR-5 and MSI-1 (P<0.05). The determination of the LI in the lesions showed that in tumours the expression of DCAMKL-1 was significantly lower (P<0.01) than in corresponding normal mucosa (Figures 7B and 8); on the contrary, we observed only a slight, not significant, reduction in MDF compared to normal mucosa (Figure 8). The difference between the labeling index in MDF and tumours was not statistically significant (P=0.10).

CD133 and ALDH1-A1 antibodies were also tested but, irrespectively of the experimental conditions used, they produced an evenly diffuse staining of the sections that indistinctly marked both the normal mucosa and the lesions, preventing us from further studying these two markers.

### Co-localization of the LGR-5, MSI-1 or DCAMKL-1 markers with nuclear β-catenin in normal mucosa, MDF and tumours (immunofluorescence experiments)

**Figure 9:**
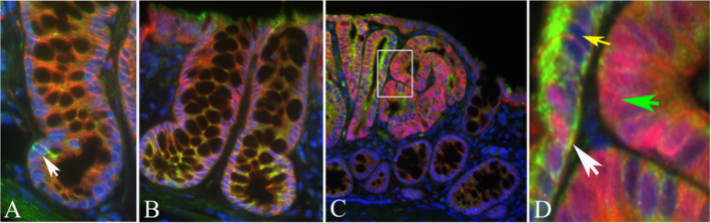
**Expression of LGR-5 in histological sections of rat colon as assessed by immunofluorescence technique.** Panel **A**: a single stained cell (white arrow) at the lower compartment of the crypt in normal mucosa. Panel **B**: multiple positive cells at the base of normal crypts. Panel **C**: LGR-5 over-expression in a preneoplastic lesion MDF. Panel **D**: Magnification of the white inset in panel C showing: LGR-5 expressing cells (yellow arrow), cells expressing β-catenin in the nucleus (green arrow) and a cell co-expressing LGR-5 and nuclear β-catenin (white arrow). Original magnification 400x.

**Figure 10:**
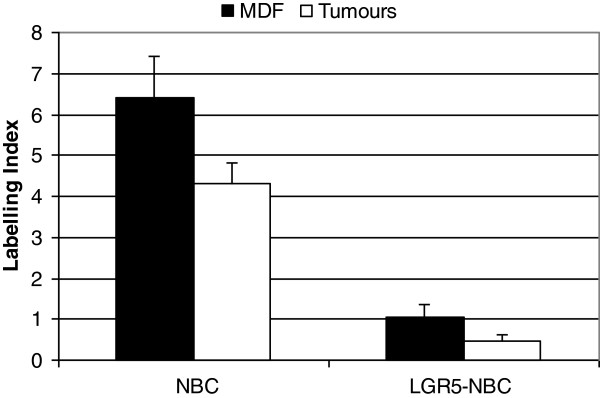
**Expression (as labelling index (LI)) of nuclear β-catenin (NBC) alone (left bars) or co-expressed with LGR-5 (right bars) in MDF (black bars) and tumours (white bars).** Values are means + SE (n=12 and 15 in MDF and tumours, respectively).

As a further step aiming to identify sub-populations of cells with the highest stemness features, we carried out co-localization experiments between each of the three markers tested (LGR-5, DCAMKL-1 or MSI-1) and nuclear β-catenin in both MDF and tumours. Regarding LGR-5, we firstly confirmed that the staining of LGR-5 with the immunofluorescence technique was similar to that observed in bright-field immunohistochemistry (Figure 9). As a matter of fact, positive cells were observed in the lower third of sporadic crypts either as single cell or multiple cells in the normal mucosa (Figure 9, panels A and B, respectively), while in the lesions (MDF and tumours), we observed an over-expression of this marker (Figure 9, panel C). Regarding β-catenin, we observed cells with nuclear β-catenin (NBC) accumulation in both MDF and tumours (Figures 9, panels C and D and 10, left group of bars), confirming our previous results [7]. In both MDF (n=12) and tumours (n=15) cells co-expressing LGR-5 and nuclear β-catenin were observed (Figure 9, panel D, white arrow). The percentage of cells showing co-localization was not statistically different in MDF and tumours (Figure 10, right bars, P=0.06). We counted 286±39 cells in MDF and 709±57 in tumours.

**Figure 11:**
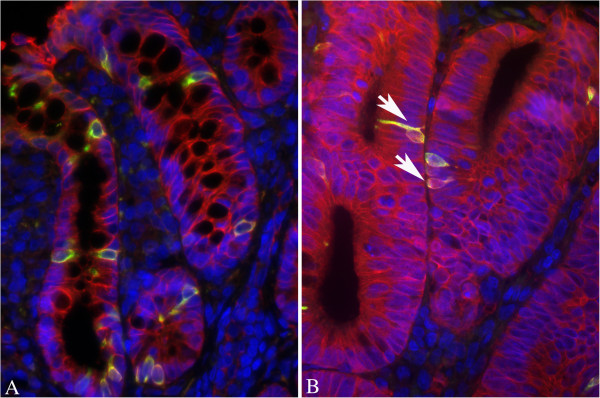
**Representative example of a co-localization experiment between nuclear β-catenin and DCAMKL-1 in histological colonic sections.** Panel **A**: normal mucosa showing DCAMKL-1 positive cells but no nuclear β-catenin. Panel **B**: tumour with cells (white arrows) co-expressing DCAMKL-1 and nuclear β-catenin. Original magnification 400x.

Co-localization experiments between DCAMKL-1 and nuclear β-catenin (NBC) were also carried out in 10 tumours and in the normal surrounding mucosa (808±128 cells in tumours and 856±180 in normal mucosa), while MDF were not available for this analysis. In this set of experiments 4.7±0.8% (means±SE, n=10) of cells expressed β-catenin in the nucleus. A representative example of DCAMKL-1 and nuclear β-catenin co-localization is reported in Figure 11. Notwithstanding the observed reduction of DCAMKL-1 positive cells in tumours compared with the normal mucosa (Figure 8), the percentage of cells co-expressing DCAMKL-1 and nuclear β-catenin was higher in tumours than in the normal mucosa (LI: 0.39±0.13 in tumours and 0.04±0.02 in normal mucosa; P<0.01 with paired t-test).

**Figure 12:**
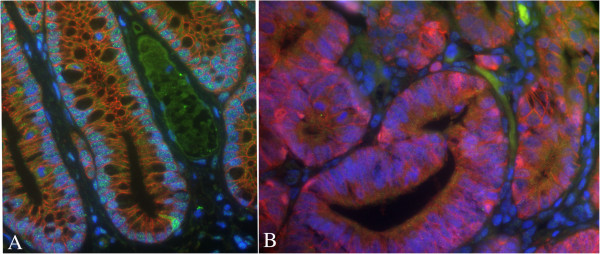
**Representative example of a co-localization experiment between nuclear β-catenin and MSI-1 in histological colonic sections.** Panel **A**: normal mucosa showing one MSI-1 positive cell at the base of the crypt but no nuclear β-catenin. Panel **B**: tumour in which many cells show nuclear β-catenin, but none express MSI-1. Original magnification 400x.

Co-localization experiments between MSI-1 and nuclear β-catenin were carried out in a small set of samples (2 MDF and 6 tumours), since, given the relatively low expression of MSI-1 in MDF and tumours, we did not expect to see cells expressing both markers. Accordingly, although we observed rare MSI-1 positive cells at the base of normal crypts also in immunofluorescence (Figure 12, panel A), none of the lesions analysed showed MSI-1 positive cells, let alone co-localizing cells (Figure 12, panel B).

### Effect of a short treatment with celecoxib (250 ppm in the diet for 2 weeks before sacrifice) on the percentage of cells expressing putative CS markers in tumours

**Figure 13:**
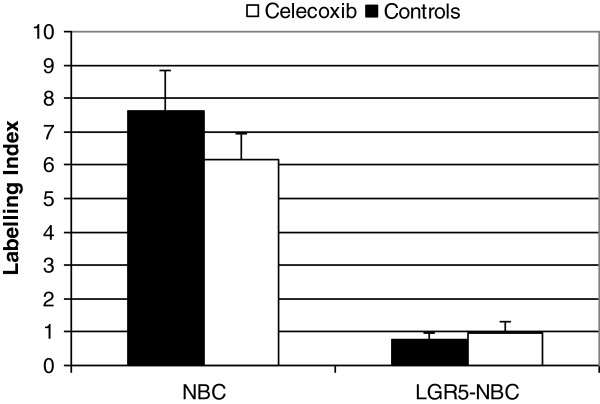
**Expression (as labelling index (LI)) of nuclear β-catenin (NBC) alone (left bars) or co-expressed with LGR-5 (right bars) in tumours from control rats (black bars) or rats treated with celecoxib (white bars).** Values are means + SE (n=10 and 10 in controls and treated rats, respectively).

Based on the above reported results showing a predominant localization at the base of normal crypts and overexpression in the precancerous lesions and tumours, we found that LGR-5 was the best putative stem cell marker among those analysed. Therefore, we considered as putative neoplastic stem cells those expressing both LGR-5 and nuclear β-catenin and we verified whether a short-term treatment with celecoxib, a COX-2 inhibitor, could reduce the number of such cells. The short period of treatment was not expected to reduce the number of colon tumours; accordingly the number of tumours/rat was 4.5±0.6 and 3.6±0.7 in controls and celecoxib-treated rats, respectively (P=0.419). Ten tumours from controls and 10 tumours from celecoxib-treated animals were analyzed (see material and methods for the detailed protocol). The results (Figure 13) show that in tumours from celecoxib-treated rats there was a slight reduction, although not statistically significant (P= 0.31) of the cells with nuclear β-catenin. However, the percentage of cells showing both LGR-5 and nuclear β-catenin was similar in the two groups (P=0.56).

## Discussion

We wanted to characterize the expression of putative markers of stem cells in the early phases of colon carcinogenesis. To this aim we studied the expression of LGR-5, MSI-1, DCAMKL-1, CD133 and ALDH1-A1 in normal mucosa, in MDF (microscopic precancerous lesions) and in macroscopic tumours (adenomas) of DMH-induced rats using immunohistochemistry. We also studied the co-localization of these markers with nuclear β-catenin, an additional proposed feature of stemness. To the best of our knowledge, this is the first study in which these markers are evaluated all together along the carcinogenesis process in this relevant model of colon cancer.

*LGR-5*, a Wnt target gene, was firstly hypothesized as a stem cell marker because of its profile of expression at the base of the crypts, compatible with that of a stem cell [21]. Further investigations with lineage–tracing experiments confirmed it as probable stem cell marker in both the small intestine and colon of mice [21]. In the present study we observed a low expression of LGR-5 in the normal rat mucosa. Only in some crypts we did observe positive cells or a diffuse staining localized mainly in the lower compartment of the crypt, although a small percent of the total labelled cells (13%) was also observed in the upper compartment of the crypt. Although we do not know the origin and nature of these rare positive cells in the luminal compartment, the low expression of LGR-5 in the normal mucosa, together with its prevalent expression at the base of the normal crypts, agrees with previous studies in humans using immunohistochemistry techniques as in the present study [28,29], and suggest that LGR-5 is a marker of stem cells also in the rat. On the other hand, the seminal papers locating LGR-5 positive cells at the base of the crypt [21,30], identified those cells by evaluating *LGR-5* gene expression (through RNA in situ hybridization) or through the expression of a chimeric protein (LGR-5-EGFP) using an antibody directed against EGFP [21,30], and thus it is difficult to compare these studies with ours.

It has been hypothesized that stem cells are more abundant in tumours than in normal mucosa [2,19] and we also observed that there was a clear overexpression of LGR-5 compared to the normal mucosa in MDF and tumours. This result, especially the overexpression in MDF, suggests that LGR-5 expression identifies putative stem cells, which are involved in the process of carcinogenesis from the very beginning, i.e. in precancerous microscopic lesions. We also observed, in agreement with previous reports in humans [21,28,31], an overexpression of LGR-5 in macroscopic tumours (i.e. the adenomas), confirming overexpression of the *LGR-5* gene that we previously reported in DMH-induced colon tumours [23].

We also studied the expression of two additional stem cell markers of colonic stem cells, i.e. Musashi-1 (MSI-1) and doublecortin and calcium/calmodulin-dependent protein kinase-like-1 (DCAMKL-1). MSI-1, firstly identified in *Drosophila* as an RNA binding protein associated with asymmetric divisions in neural progenitor cells, has been proposed by Potten and colleagues as a intestinal stem cell marker [16]. These authors reported that MSI-1 is localized in the stem compartment of the intestinal crypts and is overexpressed in small-intestinal adenomas of the *Min* mouse, a genetic model of intestinal carcinogenesis [16]. However, colonic staining in both mouse and human samples was weak [16]. Here we show that intense nuclear stained cells were present in the lower third of normal colonic crypts; in contrast, although a diffuse cytoplasmic overexpression was observed in some MDF and tumours, the nuclear staining was not observed and the majority of lesions were negative. Previous studies in normal human colon mucosa showed both cytoplasmic and nuclear staining, but the mechanisms regulating the intracellular localization of this protein are not clear [32]. Altogether, our results do not support MSI-1 as a robust cancer stem cell marker. Accordingly, in our previous transcriptomic analysis of DMH-induced colon tumours, *Msi-1* was actually downregulated compared to normal mucosa [23].

DCAMKL-1, a microtubule-associated kinase expressed in post-mitotic neurons has also been proposed as a stem cell marker [18]. However, the stem cell nature of DCAMKL-1 positive cells has been questioned [33,34]. Accordingly, DCAMKL-1 has been shown to be a specific marker of tuft, caveolated cells known for many years [33,35], but poorly characterized until now. Gerbe and colleagues studied DCAMKL-1 positive cells in mouse small intestine demonstrating that they are secretory cells expressing COX-1, COX-2 and β-endorfin [34]. Our results show that DCAMKL-1 positive cells were scattered along the entire length of the crypt in the normal mucosa, with only a slight prevalence in the lower compartment. The percentage of DCAMKL-1 cells was slightly, although not statistically significantly, reduced in MDF, while significantly diminished in macroscopic adenomas. This result is in agreement with Gerbe et al., who report a reduction of DCAMKL-1 cells in human adenocarcinomas, and with our previous results in DMH-induced tumours showing a decreased expression of *Dcamkl-1* gene [23]. In contrast, Sureban and colleagues found overexpression in human colon cancers [36].

As a further step aiming at the identification of putative cells with the most stemness features, we carried out co-localization experiments with LGR-5, MSI-1, DCAMKL-1 and nuclear β-catenin, an additional putative marker of stemness, especially in colon cancer [10,11]. Previous studies on co-localization between LGR-5 and nuclear β-catenin, performed in EGFP-creER^T2^/Apc ^flox/flox^ mice, showed that cells expressing both *LGR-5* (chimeric) and intense nuclear β-catenin staining give rise to daughter cells which still accumulate nuclear β-catenin but lose the EGFP-*LGR-5* positiveness, suggesting that the simultaneous expression of both markers identify cancer stem cells [30]. A positive correlation between LGR-5 and β-catenin expression (both in cytoplasm and nucleus) has also been reported [28], but co-localization of both markers in the same histological sections of a tumour has not been performed so far, nor has it been studied in the early phases of carcinogenesis. Here we show that in both MDF and tumours a sub-population of LGR-5 positive cells accounting for a small percentage of the lesion cells (about 1%) exhibits nuclear β-catenin staining, suggesting, on the basis of the previous considerations, that these cells could be neoplastic stem cells. The finding that the percentage of these putative neoplastic stem cells, i.e. of cells expressing the two putative markers, is unchanged between microscopic MDF and larger adenomas suggests that MDF represent the step in which stem cells overpopulation already occurs.

Co-localization between DCAMKL-1 and nuclear β-catenin shows that some cells in the tumours (about 0.4%) co-expressed DCAMKL-1 and nuclear β-catenin; this figure is similar to that observed for the co-expression of LGR-5 and β-catenin (Figure 10), but unfortunately we do not know whether these cells are the same. Therefore, also considering the lower number of DCAMKL-1 positive cells in tumours, a result not in line with the expected increase for a cancer stem marker, we can not draw any conclusions on the significance of these DCAMKL-1-nuclear β-catenin co-expressing cells.

Non-steroidal anti-inflammatory drugs (NSAIDS) are well documented as having chemopreventive activity in colon carcinogenesis [37,38]. However, besides their ability to block COX, their mechanism(s) of action at a molecular level and the specific population of cells targeted by these drugs have not been completely clarified. Accordingly, recent data show that the protective effect of NSAID may vary depending on genetic and phenotypic characteristics of the tumour [39]. Previous studies have documented a reduction of nuclear β-catenin in tumours from subjects taking daily aspirin or ibuprofen for up to 25 years [40]. Similarly, nuclear β-catenin translocation was reduced or abolished in tumours from DMH-induced rats chronically treated with various NSAIDS [41]. Accordingly, PGE_2_, a downstream product of COX-2, has been shown to enhance Wnt signaling [13] suggesting that variation in PGE_2_ within the stem cell niche may affect cell stemness properties. Recently, it has been shown that short-term pre-operative treatment with the NSAIDS indomethacin or celecoxib, (a selective COX-2 inhibitor), reduces the expression of the stem cell marker CD133 [42]. Moreover, a short treatment with the NSAID sulindac induced apoptosis in LGR-5 expressing cells and reduced nuclear β-catenin in the intestine of Min mice [43], suggesting that PGE_2_ modulates the expression of these markers and that it may be possible to down-regulate stemness properties with pharmacological treatments. To verify this hypothesis we treated tumour-bearing rats for two weeks with celecoxib to determine if the treatment was able to reduce the percentage of cells with putative stemness features which we considered those expressing both LGR-5 and nuclear β-catenin. The dose regimen of celecoxib we used has been shown to reduce COX-2 activity and colon carcinogenesis when chronically administered to AOM-induced rats [44] and is equivalent to the human dose when adjusted for the appropriate scaling factor [38]. *In vitro* studies showed that celecoxib reduces Wnt-activity in colorectal cancer cells [45]. Our results show that celecoxib causes only a slight reduction in the number of cells expressing nuclear β-catenin, which is not statistically significant; we also observed that it does not reduce cells co-expressing LGR-5 and nuclear β-catenin, indicating that, at least in these experimental conditions, the expression of these putative cancer stem markers is not affected by treatment.

## Conclusions

We characterized the expression of three putative markers of stem cells in the early phases of DMH-induced colon carcinogenesis in rats. Our results show that LGR-5, but not MSI-1 or DCAMKL-1, is up-regulated from the very early phases of colon carcinogenesis i.e. in the microscopic precancerous lesions MDF. Using immunofluorescence co-localization we identified in both MDF and tumours a sub-population of LGR-5 positive cells exhibiting nuclear β-catenin, which could be putative stem cells with the highest stemness feature driving the carcinogenesis process.

## Competing interests

The authors declare that they have no competing interests.

## Authors’ contributions

GC and APF conceived and designed the work. APF carried out the carcinogenesis experiment, the bright field and fluorescence immunohistochemistry experiments as well as the analysis of the data; he also drafted the manuscript. MS carried out part of the immunohistochemistry experiments. PD critically revised the manuscript and gave final approval of the version to be published. GC carried out the carcinogenesis experiment, collected the lesions; she also drafted the manuscript and gave the main contribution to the interpretation of data. All authors read and approved the final manuscript.

## Pre-publication history

The pre-publication history for this paper can be accessed here:

http://www.biomedcentral.com/1471-2407/13/48/prepub
